# A Qualitative Study on Youth and Healthcare Provider Perspectives on Youth Access to Sexual and Reproductive Health Services in Northern Ghana: Implications for a Peer-Led Intervention

**DOI:** 10.3390/ijerph23070915

**Published:** 2026-07-16

**Authors:** Xiaoying Zheng, Charles Timumpi Nignang, Elizabeth Holmes, Wajiha Alhassan, Zakaria Najlau, Mohammed Bahs, Breiana Brady, Nikita Kakkad, Nanki Singh, Kate Tolleson, Kwabalugu Webakura Albert, Juliana Opare-Duodu, Rayza Sison, Memunatu Chimsi, Hawa Malechi, Ana Maria Simono Charadan, Sasha Hernandez, Marie A. Brault

**Affiliations:** 1 Department of Obstetrics & Gynecology, New York University Grossman School of Medicine, New York, NY 10016, USA; xiaoying.zheng@nyulangone.org (X.Z.); sasha.hernandez@nyulangone.org (S.H.); 2 School of Nursing and Midwifery, University for Development Studies, Tamale P. O. Box TL1350, Ghana; nignangtimumpicharles@gmail.com; 3 Department of Obstetrics and Gynecology, Tamale Teaching Hospital, Tamale, Ghana; memunatuchimsi822@gmail.com (M.C.); hazumpoah@yahoo.com (H.M.); anas.amor04@gmail.com (A.M.S.C.); 4 Grossman School of Medicine, New York University, New York, NY 10016, USA; nikita.kakkad@nyulangone.org (N.K.); kate.tolleson@nyulangone.org (K.T.); 5 School of Medicine, University for Development Studies, Tamale, Ghana; alhassanwajiha@gmail.com (W.A.); zakarianajlau@gmail.com (Z.N.); webakuraalbert@gmail.com (K.W.A.); julianaopareduodu@gmail.com (J.O.-D.); 6 Institute for Excellence in Health Equity, New York University Grossman School of Medicine, New York, NY 10016, USA; mohammed.bahs@nyulangone.org (M.B.); nanki.singh@nyulangone.org (N.S.); rayza.sison@nyulangone.org (R.S.); marie.brault@nyulangone.org (M.A.B.); 7 Department of Population Health, New York University Grossman School of Medicine, New York, NY 10016, USA; breiana.brady@nyulangone.org

**Keywords:** sexual health, reproductive health, family planning services, adolescent, qualitative methods, Ghana

## Abstract

**Highlights:**

**Public health relevance—How does this work relate to a public health issue?**

- Adolescents in low- and middle-income settings, including northern Ghana, experience high rates of unintended pregnancy driven by limited access to and use of sexual and reproductive healthcare
- Early and unintended pregnancy is associated with increased risks of maternal and neonatal morbidity, making adolescent sexual and reproductive healthcare a persistent global public health priority.

**Public health significance—Why is this work of significance to public health?**

- This study identifies how informational gaps, structural barriers, and sociocultural stigma interact to limit youth engagement with sexual and reproductive healthcare.
- Using a behavioral framework (COM-B: Capability-Opportunity-Motivation-Behavior), the findings provide a structured understanding of the drivers of unmet contraceptive need among youth in a setting where evidence on peer-led sexual and reproductive healthcare interventions remains limited.

**Public health implications—What are the key implications or messages for practitioners, policymakers, and/or researchers in public health?**

- Interventions should address multiple behavioral drivers simultaneously by improving access to accurate sexual and reproductive healthcare information, reducing structural and privacy barriers, and actively addressing stigma within communities.
- Peer-led sexual and reproductive health counseling programs, co-designed with youth and grounded in local context, offer a promising strategy to increase the acceptability and uptake of services.
- Policymakers and practitioners should prioritize youth-friendly, culturally-responsive approaches to strengthen sexual and reproductive healthcare service delivery in similar settings.

**Abstract:**

Youth in northern Ghana experience high rates of unintended pregnancy due to limited access to and use of sexual and reproductive healthcare (SRH) services. Peer-led interventions are effective in increasing the youth uptake of SRH services, but limited peer-led interventions have been developed for Ghana. This study explores youth (ages 15–24 years) and clinical provider perspectives on youth SRH to inform the co-design of a peer-led SRH counseling program in northern Ghana. We conducted a qualitative study using semi-structured interviews with 22 SRH providers and 27 youth. We then conducted an inductive thematic analysis and mapped themes onto the COM-B (Capability-Opportunity-Motivation-Behavior) model. Results revealed that youth capability to make informed and confident SRH decisions is constrained by a fragmented information environment. Although access to SRH services is increasing in the region, logistical and privacy barriers limit adolescents’ opportunities to engage in care. Religious and cultural norms stigmatize youth SRH care, though support for contraceptive use is growing among more educated sectors. Youth motivation to use SRH services is driven by a desire for autonomy but undermined by concerns about contraceptive side effects and anticipated stigma. This study supports the co-development of a contextually grounded peer counseling SRH intervention in northern Ghana.

## 1. Introduction

Access to modern family planning methods enables individuals, particularly adolescent girls and women, to take control of their reproductive health and has been shown to significantly reduce maternal and child mortality [1,2]. Comprehensive sexual and reproductive healthcare (SRH), including access to contraception and abortion care or family planning, is a key component of multiple international frameworks and commitments, such as the United Nations Sustainable Development Goals, FP2030, the International Conference on Population and Development (ICPD) Program to Action, and the Guttmacher–Lancet Commission report [3,4,5,6]. Access to family planning varies widely across countries, with people in low- and middle-income countries (LMICs) experiencing substantially lower access to modern contraceptive methods and higher unmet need compared with those in high-income countries [7]. Many countries in Sub-Saharan Africa see a low uptake of family planning services due to structural, socioeconomic, and personal factors [8]. Adolescents in these countries represent a vulnerable population with a high unmet need for family planning services [9].

In Ghana, modern contraceptive use remains low, with adolescents experiencing a particularly high unmet need. According to the 2022 Ghana Demographic Health Survey (GDHS), 26% of women aged 15–49 had an unmet need for contraception, defined as women who want to delay or stop childbearing but are not using any methods. Among adolescents aged 15–19, the unmet need was much higher at 62%, more than twice the global average of 24% for this age group [10,11]. Only 32% of sexually active adolescents aged 15–24 in Ghana report using contraception [12]. Factors contributing to low uptake in Ghana include limited knowledge about contraception, stigma around adolescent sexuality, barriers to accessing comprehensive and youth-friendly services including evidence-based sexual health education, and cultural and religious norms that discourage the open discussion of sexual and reproductive health and promote abstinence or large family ideals [13,14]. In response, the Ministry of Health, together with the Ghana Health Service, the World Health Organization (WHO), and other stakeholders, have implemented programs to strengthen adolescent SRH, notably the Adolescent and Youth-Friendly Health Services (AYFHS) program, which targets young people aged 9–24 years, launched in 2010 [15].

Despite these initiatives, substantial geographic disparities persist, particularly in the northern region of Ghana, where female education is lowest, poverty is highest, and adolescent uptake of SRH services remains lower compared to other regions [16,17]. In 2024, the Academic Model Providing Access to Healthcare (AMPATH) Ghana, a global health academic partnership, conducted a landscape assessment with adolescents and young adults in Tamale, the capital of the northern region of Ghana. This landscape assessment identified the limited availability of trained providers and inadequate access to youth-friendly family planning services. Subsequently, a youth-friendly family planning counseling workshop for healthcare providers highlighted gaps in staff training, particularly in youth-friendly services and outreach, addressing misconceptions and contraceptive methods, increasing contraceptive commodity supply, integrating services, and enhancing accessibility to underserved groups. A youth panel emphasized the need for confidential, non-judgmental SRH counseling and peer-led interventions to enhance SRH literacy [18,19]. These suggestions are in line with literature that has shown that peer-led and youth co-designed interventions are key to increasing youth access to and uptake of SRH services [20,21,22].

Taken together, insights from the landscape assessment and workshop, particularly from the youth themselves, underscored the need for a peer-led SRH counseling model [18,19]. To begin to address this gap, we conducted a formative qualitative study to understand the SRH needs, preferences, and expectations of adolescents and young adults in northern Ghana to inform the co-design of a peer-led SRH counseling program. Findings may also offer insights into the current landscape of youth SRH access in northern Ghana and into the adaptation and implementation of peer-led SRH counselling programs in similar resource-constrained settings globally.

## 2. Materials and Methods

### 2.1. Study Design Overview

We conducted a qualitative study using an exploratory design informed by a constructivist paradigm. This approach was chosen to capture individual perspectives on adolescent SRH experiences and experiences with SRH service delivery in northern Ghana. Data were collected using one-on-one semi-structured interviews with adolescents and young adults aged 15–24 years old and clinicians involved in the provision of SRH services in northern Ghana, including resident doctors and junior doctors undergoing generalized postgraduate medical training as well as midwives working in family planning settings.

### 2.2. Study Context

This study was conducted in northern Ghana, where the adolescent contraceptive unmet need can reach up to 62% [10,11]. Cultural norms in the region are shaped by religious influences, particularly within a predominantly Muslim population, as well as by expectations of modesty and deeply-rooted tribal traditions [23,24,25]. The region demonstrates significant disparities in service availability across districts, with only 16% of facilities in northern areas offering comprehensive adolescent SRH services [26]. The northern region is also characterized by wide variation in educational attainment, healthcare access, and access to technology [27,28,29].

This study was conducted in partnership with Tamale Teaching Hospital (TTH), the sole tertiary teaching and referral hospital in northern Ghana, located in the Tamale Metropolitan Area, the semi-urban regional capital. TTH’s family planning unit (FPU) is staffed primarily by midwives and serves a high-volume of patients daily, providing contraceptive, abortion, and counseling care.

This research was supported by AMPATH Ghana, a global health academic partnership between NYU Grossman School of Medicine (USA), the University for Development Studies (Ghana), and Tamale Teaching Hospital (Ghana), in collaboration with the Ghana Health Service. This global public health partnership provided the programmatic infrastructure and community engagement context within which the present qualitative study was conducted.

### 2.3. Study Tool Design

Two semi-structured qualitative interview guides, one for youth and one for healthcare provider interviews, were designed by a multidisciplinary team that included researchers and clinicians with expertise in qualitative research, SRH, and adolescent-friendly care. The guides were informed by prior work including a landscape assessment focus group discussion with adolescents in the Tamale community and a youth-friendly SRH counseling workshop for healthcare providers conducted in 2024. The interview guides explored youth experiences with SRH, provider interactions with youth in SRH delivery settings, perceived barriers and facilitators to access and use, and preferences for a potential peer-based counseling intervention. The interview guides were then piloted with four youth and three healthcare providers to test for contextual relevance and refined iteratively. The final interview guides can be found in Appendix A.

### 2.4. Participant Recruitment and Data Collection

We recruited participants for this study using snowball sampling, where the initial research participants recruited additional participants from their networks. We conducted interviews with 22 healthcare providers and 27 adolescents. Among the healthcare workers, 6 (27.3%) junior doctors, 4 (18.8%) Ob/Gyn resident doctors, and 12 (54.5%) FPU midwives were included. Youth were recruited from schools and the community initially based on convenience sampling to represent a wide range of ages, educational attainment, and socioeconomic statuses. We also sought to balance the sample of youth in terms of gender. Of the youth, 15 (55.6%) were recruited from high schools, 6 (22.2%) were recruited from colleges, and 6 (22.2%) were recruited from the community (not currently attending school). Fourteen (51.9%) of the youth participants were female and 13 (48.1%) were male. Youth ranged from 15–24 years of age, with a median age of 20. Demographic information is summarized in Table 1.

Participants aged 15–17 years provided informed consent without parental consent following approval by the appropriate institutional review boards in Ghana and the United States. The waiver of parental consent was granted, as the study posed minimal risk and involved a discussion of SRH, a topic for which requiring parental consent could have created a barrier to participation and limited the inclusion of adolescents’ perspectives. To protect participants, informed consent emphasized that participation was voluntary, participants could decline to answer any question or withdraw at any time without penalty, and interviews were conducted confidentially. This approach is also consistent with the legal and policy context in Ghana, where adolescents are able to independently access family planning and other sexual and reproductive health services without parental consent. Requiring parental consent for participation in this minimal-risk study would therefore have imposed an additional barrier not present in routine SRH care and may have excluded adolescents whose perspectives are critical to understanding barriers to care. This approach is consistent with international ethical guidance, including recommendations from the Council for International Organizations of Medical Sciences (CIOMS), WHO, and the Society for Adolescent Health and Medicine (SAHM), which recognize the importance of the equitable inclusion of adolescents in research to ensure that health services and interventions are informed by the perspectives of the populations they are intended to serve [23,24,25,26,27,28,29,30,31].

All interviews were conducted between April 2025 to August 2025. Participants provided both written and oral informed consent. Interviews were all conducted in English, as the majority of youth in the Tamale metro area and clinicians, speak English [30]. Interviews lasted between 30 and 98 min and were conducted in-person, in private spaces at TTH, University of Development Studies, high schools, and in the Tamale community.

All interviews were conducted by a multidisciplinary team of researchers (W.A., C.T., A.N., X.Z.) using a semi-structured interview guide. The team included a male nurse in the TTH Department of Obstetrics and Gynecology, a U.S.-based senior female medical student, and two senior female medical students based in northern Ghana. All interviewers were close in age to the youth participants to facilitate rapport and open discussion. Youth interviews were conducted by gender concordant interviewers, given the sensitive nature of the topics discussed. The male nurse interviewer did not have a direct clinical or supervisory relationship with participating youth or staff. We stopped data collection once thematic saturation was reached, which was defined as no additional sentiments over multiple interviews [31]. Each participant was compensated per standard research compensation practices in this region.

We audio-recorded all interviews and used Otter.ai (Mountain View, CA, USA: Otter.ai, Inc., www.otterai.com) to transcribe the recordings [32]. Transcripts were then reviewed and cleaned by the research team. This study was approved by the institutional review board at New York University Langone Health (i25-00366) and the Navrongo Health Research Center Institutional Review Board (NHRCIRB640), the Ghanaian ethical review board.

### 2.5. Data Analysis

After all interviews had been transcribed and cleaned, we imported all transcripts into Dedoose 10.0.34 (Los Angeles, CA, USA: SocioCultural Research Consultants, LLC www.dedoose.com) for data management and analysis [33]. We employed a two-stage coding process utilizing thematic analysis [34]. First, one researcher (X.Z.) created two codebooks with code definitions using deductive codes based on the research question for the youth interviews and healthcare provider interviews, respectively. Inductive codes were generated through analysis of the interview data and refined through iterative reflection and team discussion. A team of researchers (B.B., M.B., M.A.B., S.H., N.K., N.S., C.T.) with training and expertise in adolescent sexual and reproductive health, youth-friendly care, implementation science, and qualitative methods independently conducted the line-by-line coding of three youth interview transcripts and three healthcare provider interview transcripts using the initial codebook. The team then reviewed and refined the codebook to ensure that it adequately captured the interview content. Two members of the coding team independently coded each transcript and met in pairs to ensure coding consistency and review discrepancies. Coding pairs were randomly assigned for each transcript. Throughout the coding process, the full coding team met regularly to discuss coding questions and resolve inconsistencies. The codebook for healthcare provider interviews was revised three times over the course of the coding process, and the codebook for youth interviews was revised twice over the course of the coding process (see Appendix B for the final versions).

**Figure 1:**
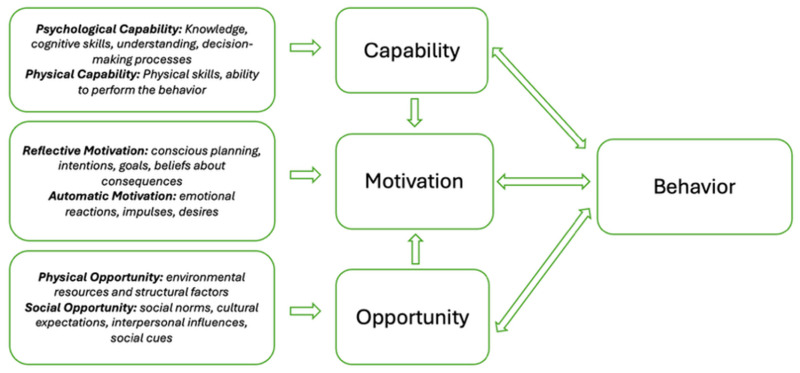
Thematic analysis results mapped onto the COM-B framework.

A theme development team (M.A.B., E.H., S.H., C.T., X.Z.) then analyzed the data in two stages. In stage 1, we generated initial themes through inductive reasoning. These initial themes were further consolidated and refined in stage 2, where we mapped emerging themes onto the Capability-Opportunity-Motivation-Behavior (COM-B) model framework. The COM-B model is a behavior change model proposing that behaviors arise from the interaction of capability, opportunity, and motivation [35]. Capability describes the knowledge, skills, and physical ability to enact a behavior. In this study, the behavior refers to adolescent use of SRH services. Motivation refers to the reflective and automatic mechanisms that activate or inhibit the use of adolescent SRH services. Opportunity describes the physical and social environment that enables the adolescent use of SRH services. A schematic of the COM-B framework is presented in Figure 1. This framework was used to organize and interpret themes by mapping identified barriers and facilitators to these three core components, as shown in Figure 1. Our approach is in line with similar qualitative analysis approaches that have been used to investigate reproductive care-seeking behavior in the past [36,37]. In stage 2, we also considered alternative frameworks, including the socioecological model, theory of planned behavior, and healthy beliefs model, though we ultimately determined that the COM-B model provided the most comprehensive framework for organizing our interview data, allowing us to capture individual, structural, and environmental factors that affected SRH uptake among youth, thus indicating areas to address in intervention development.

Member checking, a process in qualitative research in which the findings are shared with participants or relevant stakeholder groups to verify accuracy, ensure that the interpretations reflect the participants’ perspectives, and strengthen the credibility and trustworthiness of the data, was also utilized [38]. Findings were disseminated through four stakeholder-specific community forums for this purpose, including (1) department leads and researchers (*n* = 12), (2) obstetrics and gynecology (Ob/Gyn) trainees and specialists (*n* = 30), (3) FPU midwives (*n* = 9), and (4) youth community members not part of the original study (*n* = 20).

## 3. Results

Thematic analysis was conducted within the framework of COM-B [32]. This framework was used to organize and interpret themes by mapping identified barriers and facilitators to youth SRH uptake to its three core components, as shown in Figure 1. From this process, we identified four key themes, with associated sub-themes, which are summarized in Table 2.

### 3.1. Capability

#### Theme 1. Youth Capability to Make Informed and Confident Family Planning Decisions Is Limited by Perceived Inadequate Youth-Friendly Health Education and Misinformation

Youth and healthcare providers described gaps in knowledge, understanding, and skills that constrained adolescents’ ability to make informed and confident SRH decisions, largely stemming from fragmented and unreliable information environments across educational and counseling contexts. Adolescents accessed SRH information from multiple sources, including schools, healthcare workers, non-governmental organizations, social media, and peers. However, the quality and consistency of information varied widely, and myths and misinformation reduced the understanding of methods and proper use, as described in the following quotations:

*“The adolescent who is in school is privy to factual information is about family planning, but the one who is not in school is left to myths that are around and does not really have relevant information.”—High School Male #1*

*“One of our family planning clients told us that their peers advise her that immediately she has sex, she has to urinate, all the sperms will come out, so they don’t just give them accurate information about family planning.”—Family Planning Staff #2*

Youth who attended school were more likely to learn about SRH in a structured setting, though youth noted that school curricula often had gaps related to access to SRH care, contraceptive methods, STD symptoms, prevention, and testing, and more. Other information sources such as social media were seen as much more influential in shaping youth SRH beliefs; however, the accuracy of the information obtained through social media was frequently called into question by the participants.

Participants also described health system factors that further limited capability, including short consultation times, language barriers, and experiences of judgment or stigma from providers. These constraints reduced opportunities to ask questions and receive nonjudgmental guidance. Adolescents expressed a desire for accurate, comprehensive, and culturally-sensitive information from clinicians but perceived a disconnect between these expectations and their experiences, as described below.

*“Confidentiality, affordability…because part of the reason why some people will not even go is the fact that I’m going in, it’s going to be an hour judgment”—Junior Doctor #1*

Inadequate youth-friendly information and counseling environments significantly constrained adolescents’ capability to make informed and confident SRH decisions. In fact, judgment from healthcare providers was frequently cited as a barrier to youth SRH engagement.

*“Firstly, I think people should stop criticizing young girls who are going for family planning and give them the necessary knowledge that they’re supposed to know. And also health workers, some of them don’t make it easy for us or for young girls to go in for family planning.”—College Female #3*

The quality of information across sources varied considerably, with social media and peers frequently contributing to the spread of misinformation. Thus, when school-based SRH education is limited or rudimentary, and healthcare provider attitudes discourage youth from seeking care, many young people are left without reliable access to accurate information.

### 3.2. Opportunity

#### 3.2.1. Theme 2 (Physical Opportunity). While Multiple Access Points for Contraception Exist, Logistical Barriers and Privacy Concerns Restrict Adolescent Use of Family Planning Services

Participants described the high availability of SRH services across hospitals, community health centers, pharmacies, and, in some youth centers, condom vending machines. The mix of availability through health system, commercial, and community settings increased potential points of access for youth in Tamale.

*“Anywhere, because now there is [a] pharmacy around most of the community, so that’s easy for them to get it done.”—Town Female #1*

*“I’ve had a friend who usually comes to deliver me condoms. He said there was a machine at a youth home [provides vocational training and recreational activities for young people]. When you put in money, and then you press, it rolls, and then it brings out a condom.”—High School Male #2*

However, adolescents, particularly in rural areas, continued to face logistical barriers, including transportation difficulties, long distances, unreliable supplies, and cost, which constrained their ability to obtain contraception, as described in the following quotations.

*“The cost actually is also part… the [contraception] commodities have increased in prices … [patients] would tell me, oh, initially I thought it was this price, but now I don’t have the money to afford it.”—Family Planning Staff #1*

*“I had to travel from my village to another village, because where I’m coming from, there’s no clinic or hospital there.”—College Female #3*

Privacy concerns within healthcare facilities further discouraged use. FPUs were often located in highly visible areas.

*“Most of them… can easily walk to any facility and get them, but because of the stigma… most of them will not willingly walk to a facility because, ‘Oh, someone else will see me. This person will see me. They will tell my parents.’”—Ob/Gyn Doctor #1*

*“Most of the teenagers don’t like to go to the hospital because if they go there, maybe they might see someone their father’s age, so that person will not be comfortable there.”—High School Female #5*

Thus, while physical access to SRH services has expanded, persistent structural and environmental barriers continue to restrict adolescents’ opportunity to engage with SRH services.

#### 3.2.2. Theme 3 (Social Opportunity). Adolescent Access to Family Planning Is Shaped by Social Norms and Relationships, Where Widespread Stigma Poses Barriers Alongside Emerging Pockets of Community Support

Participants described social norms within the community that framed adolescent SRH engagement as immoral or promiscuous, discouraging service use and reinforcing expectations of youth abstinence outside of marriage.

*“[People in the community] always make me feel so bad because they will just think you’re a bad person, or going for it [family planning] makes you a bad person.”—High School Female #1*

*“Some are shy of it [family planning]… they feel like when you’re on this kind of thing [contraception], you’re a bad girl or a bad boy… because of how people think of them, they hide it and refuse to do it.”—College Female #1*

This stigma came from the belief that family planning engagement is closely tied to premarital sex, which was seen as shameful and led to further stigmatization of SRH use. These beliefs were prevalent among many community members, which in turn led to widespread stigma surrounding SRH, leading to judgment and shaming.

*“Because of the social, cultural aspects of this community, we just have this perception that sex is just confined to marriage and premarital sexual activities is frowned upon. People will think they are tagged as bad people and all that. So it drives them away from family planning services”—Junior Doctor #2*

While youth described parents and community elders as judgmental toward contraceptive use, the opinions of peers and romantic partners were perceived to be more mixed. Peers and partners were influential in youth engagement in SRH, serving as sources of information and support.

*“Your friend might have it [contraceptive], or your friend might even go and buy it for you, if you feel shy, to get to the pharmacist. Or your friend might even have sachet of that (contraceptive) and give you some”—College Male #1*

*“He [partner] was the one who said I should allow him to go and buy for me”—High School Female #2*

When discouragement occurred among peers and partners, it was often rooted in broader social and cultural stigma or interpersonal dynamics informed by assumption and misinformation, with some young people reporting being labeled as “immoral” for using SRH.

*“Her boyfriend… he was like, why would you go in for implant when we don’t do such things [have sex]? What’s your reason for going in for implant if you’re not cheating on me?”—College Female #2*

*“Some friends believe that family planning methods aren’t good socially, and that’s anyone who uses it is considered a bad person… they always say that friend is a bad person”—Town Male #1*

Participants also noted growing recognition in the community of the benefits of delaying pregnancy, specifically with regards to pursuing education and professional advancement prior to becoming pregnant, which has increased the social acceptability of contraceptive use among more educated community members.

*“The educated ones, tend to have so much knowledge about family planning, it’s significant. So, they patronize family planning a lot based on what they read in books or what they are taught in class…because of education, they actually delay childbearing.”—Ob/Gyn Doctor #2*

*“[Girls] will say, let me use it for now, since I can’t abstain from sexual activities, so let me go for the family planning. If I go and do it, I will not cause harm to my education.”—High School Male #3*

Adolescents’ social opportunities to use SRH services reflect a tension between persistent societal stigma and emerging normative support for delayed childbearing within their immediate communities, especially among youth who were in school. Importantly, even with emerging support, youth continued to report discouragement from their immediate communities, including peers, partners, and family.

### 3.3. Motivation

Theme 4. Youth Motivation to Engage in Family Planning Is Driven by a Desire for Autonomy, and Further Shaped by Stigma, Fear, Privacy, Risk Perception, and Information Appraisal

Participants described adolescents’ motivation to use SRH as strongly rooted in a desire for bodily autonomy and control over their futures.

*“I’m in senior high school… doing family planning, it can prevent that child coming and making me finish my education before I will now go and give birth again”—High School Female #4*

*“The adolescents themselves make a decision, because at the end of the day, they don’t openly discuss with parents. When they feel the need for family planning, they go for it”—High School Male #1*

In this way, SRH was framed as a tool for young people, especially young women, to maintain control over their future opportunities. As SRH is mostly seen as a female-oriented healthcare service, much of the discussion centered around young women’s motivations for using SRH.

Youth motivation to access SRH services was also shaped by concerns about social judgment and stigma, which were often rooted in the deeply religious context of the community. Participants described how religious norms and community attitudes discouraged youth engagement with SRH.

*“The natives of this place are mostly Muslims, and it appears as that their religion frowns against family planning, so it makes it difficult for people of my age [22].”—High School Male #1*

*“I know that if you do family planning, maybe the time that you want to give birth, you will not be able to give birth, and your womb can be closed because of the family planning. People say it’s a sin to do family planning—High School Female #3*

Uncertainty about side effects and method visibility, which were linked to knowledge gaps previously described, also reduced willingness to initiate or continue use. Youth reported fear of side effects such as weight changes, unpredictable bleeding, acne, and infertility in the future.

*“For adolescent, the first thing on the list is I will get very fat and break out, get lots of acne when I start doing family planning, and then as they age, those in their 20s and above, the misconception is, once you do family planning, when you’re ready to have kids, it’s very hard. You may have fertility issues, or you would keep bleeding, you would over bleed, and it’s so uncomfortable, you feel sick all the time, and all that.”—Junior Doctor #3*

*“You can do it in a way that none of your friends or someone else suspects; like the implant, by all means, someone will one day know [that you are using it], but the condom and contraceptive, they will never know, that is between you and your partner”—College Female #1*

Supportive peers and partners and nonjudgmental, confidential healthcare encounters were described as important facilitators of youth motivation to accessing family planning that counteracted stigma, strengthened adolescents’ sense of autonomy, and promoted more positive emotional motivation toward SRH.

*“Most health professionals, they don’t frown on that [family planning]. They make it readily accessible because they have the knowledge and benefits, so they don’t restrict that…from the peers”—College Male #1*

*“Actually, it depends on the type of friends you have. Some youth, at my age, they are already exposed to some of those things (family planning) and some are not. So, if the person is exposed, I think the person will convince you but if the person is not exposed, maybe they will discourage you”—College Female #3*

Overall, adolescents’ motivation is shaped by an interplay between their desire for autonomy, fear of stigma leading to emotional barriers, and personal information appraisal shaped by misinformation, alongside the protective influence of supportive relationships and nonjudgmental care.

## 4. Discussion

This formative qualitative study explored the SRH needs, preferences, and expectations of adolescents and young adults in northern Ghana to inform the co-design of a community-based peer-led SRH counseling program in Tamale. Our findings underscore that youth engagement with SRH services is determined by a combination of structural, social, and environmental factors that can be conceptualized using the COM-B framework as we work toward intervention design. While many of the barriers identified have been documented in prior literature, this study centers youth perspectives in northern Ghana, which is historically understudied, to translate these insights toward a co-designed intervention. By mapping barriers and facilitators onto the COM-B framework, we identified modifiable behavioral and structural determinants that can inform peer-led intervention design. These findings directly affect the co-design of our intervention, providing an example of how qualitative research can be translated into intervention development for youth SRH access in a resource-limited setting. Our study contributes to a small but growing body of literature using a participatory co-design approach to develop adolescent SRH interventions by integrating behavioral analysis through the COM-B framework to guide intervention development [33,34,35,36].

### 4.1. Capability

Youth described a fragmented, misinformation-prone information environment characterized by peer-based knowledge transmission, reliance on social media, persistence of myths and misinformation, as well as limited formal or comprehensive SRH education. Participants emphasized that misinformation often circulates unchecked within social networks, which reinforces the fear of side effects and uncertainty about contraceptive safety. Limited youth-friendly health education further limits youth ability to make informed and confident SRH decisions.

These findings align with prior studies examining youth access to SRH in Ghana, which document a lack of knowledge, limited formal SRH education, and attitudes of service providers, as well as pervasive misinformation, as barriers to youth access to SRH services [14,37,38,39]. Similar barriers have been documented in systematic reviews of youth SRH access in Sub-Saharan Africa (SSA) and LMICs [13,40]. Thus, our current study reinforces the persistent role of misinformation and structural barriers in shaping youth SRH access. Our present study further illustrates how misinformation is shared and sustained within youth communities, particularly through informal channels. This underscores the need for SRH counseling models that leverage the social and informational spaces youth already engage in, while also ensuring that counselling curricula are evidence-based.

### 4.2. Opportunity

Youth and providers noted logistical barriers such as transportation challenges, costs, and privacy concerns that restricted physical opportunity to access SRH services. Of note, participants described expanding SRH availability in the northern region of Ghana, though this expansion did not fully address concerns around confidentiality, discretion, or logistical barriers. Even when health facilities were available, youth reported fear of being recognized by other community members as a deterrent to seeking care. Our study highlights the persistence of social and structural barriers that continue to limit their ability to access care.

Persistent sociocultural barriers such as religious discouragement and community stigmatization of youth sexuality limited youth social opportunity to access SRH, though emerging community support for delayed childbirth increased their social opportunity, especially among more educated community members. Although peers and partners were generally supportive of youth SRH use, they could also serve as strong barriers when they opposed it. Because many young women relied on male partners for financial support to obtain SRH services, male partners held substantial influence over contraceptive decisions. This finding is in line with research across multiple contexts finding that financial dependence on male partners consistently reinforces gendered power imbalances that limit young women’s autonomy in sexual and reproductive health, constraining their ability to access services and make independent contraceptive decisions [41,42,43,44]. Across diverse settings, evidence shows that male partner influence through financial control, social pressure, and relational dynamics undermines reproductive agency, highlighting the need for interventions that address economic empowerment, partner dynamics, and broader gender norms [41,43,44,45].

Our findings are consistent with prior studies across Ghana, noting logistical barriers and pervasive social stigma stemming from cultural and religious beliefs [14,37,38]. Similarly, logistical barriers were also noted across studies based in SSA and other LMICs [13,40]. However, our study documents a perceived expansion of SRH services in northern Ghana, a trend that has rarely been reported in the existing literature, which typically emphasizes that persistent unmet need is due to a lack of available contraceptive options [8,9]. Our study further pointed out that the availability of services alone is insufficient to encourage youth engagement and reduce unmet need. Perceived privacy and social acceptability are also important determinants of SRH access and engagement. Thus, community-based peer-led counseling approaches may be well-suited to addressing these constraints, reducing the visibility of SRH engagement and offering trusted, youth-friendly counselors [20,21,22].

### 4.3. Motivation

Youth motivation to use SRH was shaped by a desire for autonomy in reproductive decision-making, as participants expressed the importance of controlling pregnancy timing and protecting educational goals. These findings are consistent with prior research, primarily among adult women, illustrating the challenges around negotiating autonomy in a restrictive social environment [46,47]. However, this motivation was tempered by social consequences, fear of side effects, and uncertainty regarding misinformation, which has also been reported in previous studies [13,38,40,48,49]. In the present study, youth described navigating tensions between their personal goals and anticipated judgment from family, partners, peers, and community members. This tension highlights the importance of interventions that actively support youth autonomy, confidence, and self-efficacy in engaging in SRH.

Overall, our findings are consistent with previous studies examining contraceptive access in Ghana, which report interacting individual, social, and structural factors such as fear of judgment, stigma, religious and cultural beliefs, social influences, as well as inequities related to education, health system access and quality [13,18,19,38,40,46,47,48,49]. In addition, our study underscores the role of autonomy in youth motivations to use SRH services, a factor that has been underexplored in relation to adolescent SRH globally.

### 4.4. Implications for Co-Design of a Peer-Led Model

Our findings highlighted the complex and nuanced forces shaping youth engagement to SRH at our study site, as well as key barriers that can be addressed with a structured peer-led intervention, including knowledge gaps, misinformation, shame and fear surrounding SRH, and anticipated stigma from healthcare providers. They also underscore the need for widely accessible, multi-platform peer counseling, given that transportation and privacy were frequently reported barriers.

This formative study was conducted to inform the co-design of a peer-led SRH intervention in the Tamale area. By centering perspectives from youth directly affected by barriers to SRH care alongside healthcare workers familiar with the current service landscape, our findings offer insight into how SRH services may be structured to better align with youth priorities and expectations.

In the capability domain, we found that the current SRH information environment is fragmented, and peers serve as a key source of information, suggesting that peer counselors may be a natural and appropriate point of information transmission. In the opportunity domain, both physical and social barriers limit youth ability to access SRH services, highlighting the importance of privacy, accessibility, and confidentiality in intervention design. In the motivation domain, youth expressed interest in using SRH services but described being discouraged by stigma and the structural barriers noted above, suggesting that motivation alone is not sufficient to drive service uptake in this context.

Together, the COM-B domains show that a peer-led SRH intervention is well-aligned with the behavioral determinants identified in this study and highlight specific elements that should be considered in its design, including confidentiality, accessibility, and trust.

Additionally, our study incorporates perspectives from multiple stakeholders, including healthcare workers and youth. During member checking procedures, FPU midwives emphasized that the unit had already implemented several measures to protect patient privacy. This observation highlights a tension between youth perceptions of persistent privacy concerns and SRH clinicians’ beliefs that these issues have been addressed to the extent possible within the clinical setting. These differing perspectives underscore the importance of engaging diverse stakeholders when evaluating barriers to care, as experiences and perceptions may vary substantially across groups.

These findings will inform future participatory processes aimed at designing and implementing a structured peer-led SRH program. Additionally, this work may also inform adolescent SRH programming in other low-resourced, socio-culturally conservative settings. The barriers identified in the present study are not unique to this region and have been widely documented in similar contexts. Thus, the design and implementation of a structured peer-led SRH program in this context may provide insight into the feasibility, acceptability, and effectiveness of a peer-led model in comparable contexts.

### 4.5. Implications for Structural and Social Interventions

While peer-led interventions may address several interpersonal and knowledge-based barriers identified in this study, our findings also demonstrate that broader structural changes are needed to improve youth access to SRH services. Participants described barriers spanning educational systems, healthcare delivery, community norms, and logistical constraints, suggesting that no single intervention is likely to fully address existing inequities.

These findings support the need for complementary interventions, including comprehensive SRH education, continued provider training in youth-friendly care, improved transportation and service accessibility, and community engagement initiatives that reduce stigma surrounding youth SRH service engagement. The breadth of barriers identified suggests that adolescent SRH access is shaped by interactions across multiple levels of the health system and its surrounding social context, rather than by healthcare services alone. As such, peer-led interventions may be most effective when implemented as part of a broader adolescent-responsive system that aligns service delivery, school-based education, community engagement, and supportive local policies to reduce barriers to care [6,7,50,51].

Our findings also highlight how gender and social power dynamics shape adolescents’ access to SRH services. Although many barriers were described as community-wide, young women often bore disproportionate social consequences for seeking contraception or other SRH services, reflecting prevailing gender norms surrounding sexuality, premarital sex, and reproductive decision-making. Participants described concerns about social judgment from peers, families, and community members, and fears of being perceived as sexually active, suggesting that decisions to seek care are embedded within broader social and gendered expectations rather than being solely individual choice. These findings underscore that improving adolescent SRH access requires not only increasing service availability, but also addressing the gendered social and structural conditions that constrain young people’s reproductive autonomy [42,52].

A rights-based approach to adolescent SRH recognizes young people’s ability to make informed decisions about their sexual and reproductive health while emphasizing equitable access to confidential, acceptable, and youth-responsive services [53]. Our findings suggest that peer-led interventions may help adolescents navigate interpersonal barriers; however, they should be implemented alongside efforts that address inequitable gender norms, engage parents and communities, and strengthen providers’ capacity to support adolescents’ reproductive rights without judgment. Such approaches may be particularly important in settings where social and gender norms continue to influence whether, when, and how young people seek SRH care [51,54].

### 4.6. Strengths and Limitations

Our study focused specifically on the northern region of Ghana, which has the lowest levels of SRH uptake in the nation [16]. To our knowledge, only four studies have specifically evaluated youth SRH access in this region to date [37,39,55,56]. Evaluating youth SRH access in this setting is critical to informing interventions that are responsive to the needs of this population.

Strengths of this study include its focus on youth voices, as well as qualitative design. In this, it provides context-specific insights from narrative experiences, relevant to intervention design. The focus on the northern region of Ghana addresses a gap in the literature, and the inclusion of both youth and provider perspectives strengthens the reliability of the findings.

There were also several limitations. This study did not include youth from rural communities or youth who did not speak fluent English, which further limited the representativeness of the sample. While we initially included Dagbani-speaking youth in our interviews, our team ultimately did not have sufficient capacity to conduct and translate interviews in Dagbani. Thus, the participants were predominantly youth who had access to formal education, and their experiences may not reflect those of out-of-school youth or those with lower levels of educational attainment. These populations may face different barriers to accessing SRH information and services, including greater structural, linguistic, or socioeconomic challenges. As a result, our findings may not be generalizable to all youth in northern Ghana, particularly those living in more rural or underserved communities. Additionally, our peer-led intervention design may be less responsive to the needs and preferences of out-of-school youth who primarily speak Dagbani. Provider interviews were restricted to TTH due to logistical constraints, which may limit generalizability to other healthcare settings in the region. As this study was focused on sensitive topics, responses from the youth and providers may also have been influenced by social desirability bias. However, despite these limitations, these insights serve as an initial framework for the development and future implementation of a co-designed, peer-led SRH intervention.

## 5. Conclusions

Youth engagement with SRH services in northern Ghana is shaped by interacting informational, social, and structural barriers rather than a single dominant constraint. Knowledge gaps and misinformation, alongside shame, fear, and anticipated stigma from healthcare providers, limit the use of available services. These individual and social challenges are compounded by structural barriers such as transportation constraints and lack of privacy, which further restrict access even among motivated youth.

Despite these barriers, participants expressed clear interest in accessing SRH services motivated by a desire for reproductive autonomy, suggesting that low utilization reflects constrained opportunity and capability rather than lack of motivation. The prominence of peer networks as a trusted source of information further underscores the potential role of peers in addressing these gaps.

This study utilized the COM-B framework to identify key targets for intervention design and highlights the potential of a peer-led SRH counseling model to increase SRH engagement. These findings are being used to inform the co-design of a peer-led intervention tailored to the needs and priorities of youth in northern Ghana. Beyond intervention design, our findings also point to the need for broader structural changes such as strengthened comprehensive SRH education, continued provider training in youth-friendly care, improved geographic and practical accessibility of services, and community engagement to reduce stigma. Given the influence of gender norms and power dynamics, broader efforts to promote gender equity and normalize youth engagement with SRH services will likely be essential for sustained improvements in access.

## Figures and Tables

**Table 1:** Demographic information of the interviewees.

	Youth	Healthcare Providers
**Sample**	27	22
Female (%)	14 (51.9%)	16 (72.7%)
Male (%)	13 (48.1%)	6 (27.3%)
Median Age	20	
Age Range	15–24	
Recruited from		
High School (%)	15 (55.6%)	
College (%)	6 (22.2%)	
Town (%)	5 (22.2%)	
Healthcare Provider Role		
Junior Doctor (%)		6 (27.3%)
Ob/Gyn Doctor (%)		4 (18.8%)
Family Planning Unit Midwife (%)		12 (54.5%)

**Table 2:** Summary of key themes and sub-themes mapped to COM-B domains.

COM-B Domain	Theme	Sub-Themes
Capability	Theme 1: Limited ability to make informed SRH decisions due to inadequate education and misinformation	- Inconsistent information sources - Myths and misconceptions - Gaps in school-based education - Limited youth-friendly counseling - Provider judgment and communication barriers
Opportunity (Physical)	Theme 2: Access constrained by logistical and privacy barriers despite service availability	- Multiple service points - Cost, distance, and transport barriers - Supply issues - Lack of privacy - Fear of being seen
Opportunity (Social)	Theme 3: Stigma and social norms restrict SRH use, alongside emerging support	- Community stigma and moral norms - Parental and societal judgment - Peer/partner influence (supportive and discouraging) - Increasing acceptance linked to education
Motivation	Theme 4: Motivation shaped by autonomy, stigma, fear, and perceived risks	- Desire for autonomy and future control - Stigma and religious norms - Fear of side effects and infertility - Preference for discreet use - Influence of relationships and supportive care

## Data Availability

The raw data supporting the conclusions of this article will be made available by the authors on request.

## Abbreviations

The following abbreviations are used in this manuscript:

SRH	Sexual and reproductive healthcare
ICPD	International Conference on Population and Development
LMIC	Low- and middle-income countries
GDHS	Ghana Demographic Health Survey
WHO	World Health Organization
AYFHS	Adolescent and Youth-Friendly Health Services
AMPATH	Academic Model Providing Access to Healthcare
TTH	Tamale Teaching Hospital
FPU	Family Planning Unit
CIOMS	Council for International Organizations of Medical Sciences
SAHM	Society for Adolescent Health and Medicine
COM-B	Capability-Opportunity-Motivation-Behavior
Ob/Gyn	Obstetrics and gynecology
SSA	Sub-Saharan Africa

## Appendix A

### Youth and Staff Interview Guides

#### Interview Guide for Youth Concerning Contraceptive Access, Counselling, and the Potential for Peer Counselling/Champions to Improve Youth-Friendly Contraception Access in Northern Ghana

Introduction: *Thank you for taking the time to speak with us today. As described in the consent information, we are interested in hearing your thoughts on family planning access and counselling in this community, and your thoughts on a new program to provide peer family planning counselling and support. We are interested in learning how family planning access and counselling currently works. Specifically, we would like to hear how people your age think about family planning—not just how you access it, but how you feel about it, who you talk to about it, how it fits into your relationships and life. Your ideas will help us think about how a new program that could help people like yourself, and ways the program could be improved. The interview questions are open-ended, and we invite your reflections, positive or negative, on existing and future programs.*

*We would like your permission to record this interview. This lets us listen carefully to you rather than taking notes and ensures that we will accurately capture our conversation. All information will be kept strictly confidential and no identifying information about you is included on the transcript. Digital files with audio-recorded material will be deleted as soon as the transcripts have been reviewed for accuracy. If at any point you would like me to turn off the recorder, please let me know. You are free to decline to participate, to end our interview at any time for any reason, or to choose to skip any question.*

#### Obtain Verbal Consent

Do you agree to participate in this interview and for it to be recorded? (Yes/No)

1. Please tell us a little bit about yourself.
  - Age
  - Education
    - Currently in school
    - When they left school if not currently in school (or if they never attended school, why that was)
  - Occupation (if not in school)
  - Family—who they live with, etc.
  - Things you enjoy doing for fun
2. We want to hear your thoughts on family planning (the things people use to prevent pregnancy) access in counselling in this community.
  - What sorts of family planning methods are available to people your age in this community?
  - What sorts of contraceptive methods do people your age in this community use?
    - What do you think are the most common forms of family planning people your age use?
      1. Why do you think it is so common?
    - Why do you think most people your age would want to use family planning or contraception?
  - Besides clinics and health workers, where else do young people get information about family planning?
    - Friends? Social media? School? Family members?
    - Tell us about any stories or beliefs you’ve heard about family planning that you think are common among people your age?
  - What makes it easier for people your age to access and use family planning?
    - Knowing what’s available
    - Logistics (cost, transportation)
    - Support from others (and probe for who these people are—friends, romantic partners, parents, healthcare providers)
    - Attitudes toward family planning (acceptable to use, easy to negotiate use of condoms or other methods)
  - What makes it harder for people your age to access and use family planning?
    - Knowing what’s available
    - Logistics (cost, transportation)
    - Lack of support from others (and probe for who these people are)
    - Some people have concerns about side effects or what family or partners might think. Did these kinds of things ever come up for you or others you know?
    - Attitudes toward family planning (and probe for who these people are—friends, romantic partners, parents, healthcare providers)
    - Who usually helps decide whether someone your age can use family planning?
  - How, if at all, do other parts of young people’s lives (school, relationships, social expectations, work, other aspects) influence their beliefs on family planning
3. We want to hear about your experiences with family planning. Have any of your friends used any family planning methods? Which methods did they use?
  - What do you think made it easier for your friend to use the method
    - Knowing what’s available
    - Logistics (cost, transportation)
    - Support from others (and probe for who these people are—friends, romantic partners, parents, healthcare providers)
    - What are the attitudes toward your friends using family planning? (for example, acceptable to use, easy to negotiate use of condoms or other methods)
    - Ease of continuing the method
  - What made it harder to use the method?
    - Knowing what’s available, knowing the side effects
    - Logistics (cost, transportation)
    - Support from others (and probe for who these people are—friends, romantic partners, parents, healthcare providers)
    - Challenges continuing the method
4. Can you share any family planning methods that you/your romantic partner(s) have ever used? If you/your romantic partner(s) have never used a family planning method, please share why that is.

IF YES:
  - What made it easier to use the method?
    - Knowing what’s available
    - Logistics (cost, transportation)
    - Support from others (and probe for who these people are—friends, romantic partners, parents, healthcare providers)
    - What are the attitudes toward you using family planning? (for example, acceptable to use, easy to negotiate use of condoms or other methods)
    - Ease of continuing the method
  - What made it harder to use the method?
    - Knowing what’s available, knowing the side effects
    - Logistics (cost, transportation)
    - Support from others (and probe for who these people are—friends, romantic partners, parents, healthcare providers)
    - Challenges continuing the method
IF NO:
  - If you were to use a family planning method, what are some factors that make it easier to access or use family planning?
    v. Knowing what’s available
    vi. Logistics (cost, transportation)
    vii. Support from others (and probe for who these people are—friends, romantic partners, parents, healthcare providers)
    viii. What are the attitudes toward you using family planning? (for example, acceptable to use, easy to negotiate use of condoms or other methods)
    ix. Ease of continuing the method
  - What might make it harder to use the method?
    x. Knowing what’s available, knowing the side effects
    xi. Logistics (cost, transportation)
    xii. Support from others (and probe for who these people are—friends, romantic partners, parents, healthcare providers)
    xiii. Challenges continuing the method

5. Thank you for sharing that information! Now, we would like to discuss ways to improve family planning access and use in this area. There is growing evidence that youth-friendly family planning services can significantly improve both knowledge and uptake of contraceptive methods among young people. These services often include features like confidential visits, dedicated counseling spaces to ensure privacy, low or no-cost contraceptives (sometimes available over the counter), peer educator involvement, mobile or digital platforms for discreet access to information, and integration of counseling into school curriculums. If we waved a wand, and put you in charge of things here, what would your ideal family planning set-up for young adults and adolescents be?
  - Probe for location of counselling and accessing the method (schools, clinics, pharmacies, online, other)
  - Probe for who would deliver the counselling and the characteristics of that person
  - Probe for the content (what topics should be included)
  - Probe for the format of the counselling and education (one-on-one discussions, group discussions, lectures, videos, etc.)
  - Probe for how linkage to access the method would happen
  - What kind of person would young people trust to talk about family planning?
6. This is our last set of questions. We are going to describe a program that we are thinking about starting here, and we would like to hear what you think would work, or not work. We want to make this program work for young people like you, so please feel free to share.
  - We have heard that young people do not always feel comfortable going to the clinic or hospital and speaking to the adults in charge about family planning. So, we would like to train someone your age to counsel youth on family planning. That counsellor or champion would share information with other young people about the different methods and where they can access them. They could also go with the young person to the clinic to help them get linked with one of the providers.
    - What do you think about this?
    - How, if at all, would you change this to better fit with your ideal?
    - How should the peer counsellor provide information? Pamphlets, or electronically?
    - How should youth contact the peer counsellor?
    - Where would be the best environment or place to talk to a peer counsellor (clinic, school, over text message, WhatsApp, somewhere else)?
    - If the young person decides that they want to try family planning, how should the peer counsellor support them in this?
7. Thank you for your time and expertise. Before we end, is there anything else that we didn’t ask that you think would be helpful for us to know about family planning for youth, or our new program?

#### Interview Guide for Family Planning Staff in Tamale (And Surrounding Areas)

Introduction: *Thank you for taking the time to speak with us today. As described in the consent information, we are interested in hearing your thoughts on family planning access and counselling in this community, and your thoughts on a new program to provide peer family planning counselling and support. We are interested in learning how family planning access and counselling currently works, how this new program might fit in, and your recommendations for this new program. The interview questions are open-ended, and we invite your reflections, positive or negative, on existing and future programs.*

*We would like your permission to record this interview. This lets us listen carefully to you rather than taking notes and ensures that we will accurately capture our conversation. All information will be kept strictly confidential and no identifying information about you is included on the transcript. Digital files with audio-recorded material will be deleted as soon as the transcripts have been reviewed for accuracy. If at any point you would like me to turn off the recorder, please let me know. You are free to decline to participate, to end our interview at any time for any reason, or to choose to skip any question.*

#### Obtain Verbal Consent

Do you agree to participate in this interview and for it to be recorded? (Yes/No)

1. Please tell us a little bit about yourself.
  - Age
  - Job title
  - In what clinical capacity do you provide family planning services (sample probing questions: counseling at ANC, treatment of STDs, abortion services, working at the FP Unit)
2. We want to hear your thoughts on family planning access for adolescents and young adults (13–24 years old) and counselling in this community.
  - What sorts of contraceptive methods do adolescents and young adults in this community tend to use or prefer?
  - Besides clinics and health workers, where else do young people get information about family planning?
    - Friends? Social media? School? Family members?
    - Tell us about any stories or beliefs you’ve heard about family planning that you think are common among adolescents and young adults in this area?
  - What makes it easier for adolescents and young adults to access and use family planning?
    - Knowing what’s available
    - Logistics (cost, transportation)
    - Support from others (and probe for who these people are—friends, romantic partners, parents, healthcare providers)
    - Attitudes toward family planning (acceptable to use, easy to negotiate use of condoms or other methods)
  - What makes it harder for adolescents and young adults to access and use family planning?
    - Knowing what’s available
    - Logistics (cost, transportation)
    - Lack of support from others (and probe for who these people are)
    - Some people have concerns about side effects or what family or partners might think. Did these kinds of things ever come up for you or others you know?
    - Attitudes toward family planning (and probe for who these people are—friends, romantic partners, parents, healthcare providers)
    - Who usually helps decide whether a youth or young adult (13–24) can use family planning?
  - How, if at all, do other parts of young people’s lives (school, relationships, social expectations, work, other aspects) influence their beliefs on family planning
3. Thank you for sharing that information! Now, we would like to discuss ways to improve family planning access and use in this area. There is growing evidence that youth-friendly family planning services can significantly improve both knowledge and uptake of contraceptive methods among young people. These services often include features like confidential visits, extended hours of operation, dedicated counseling spaces to ensure privacy, low or no-cost contraceptives (sometimes available over the counter), peer educator involvement, mobile or digital platforms for discreet access to information, and integration of counseling into school curriculums. If we waved a wand, and put you in charge of things here, what would your ideal family planning set-up for adolescents and young adults be?
  - Probe for location of counselling and accessing the method
  - Probe for who would deliver the counselling and the characteristics of that person
  - Probe for the content of the counselling and education
  - Probe for how linkage to access the method would happen
  - What kind of person would young people trust to talk about family planning?
4. This is our last set of questions. We are going to describe a program that we are thinking about starting here, and we would like to hear what you think would work, or not work.
  - We have heard that young people do not always feel comfortable going to the clinic or hospital and speaking to the adults in charge about family planning. So, we would like to train someone in the adolescent and young adult age group, or slightly older (maybe 16–24 years old) to counsel other youth on family planning. That counsellor or champion would share information with other young people about the different methods and where they can access them. They could also go with the young person to the clinic to help them get linked with one of the providers.
    - What do you think about this?
    - How, if at all, would you change this to better fit with your ideal?
    - How should the peer counsellor be trained?
    - What characteristics should the peer counsellor have?
      1. What do you think about having slightly older (16–24 yrs) counselors vs. including younger adolescent (13–24) counselors as well?
    - How should the peer counsellor provide information? Pamphlets, or electronically?
    - How should youth contact the peer counsellor?
    - If the young person decides that they want to try family planning, how should the peer counsellor support them in this?
    - How, if at all, would you want your team to work with this peer counsellor?
5. Thank you for your time and expertise. Before we end, is there anything else that we didn’t ask that you think would be helpful for us to know about family planning for youth, or our new program?

## Appendix B

### Codebooks for Youth and Staff Interviews

Scheme Interview Codebook

**Code**	**Definition**
FP Provider Characteristics	Characteristics of TTH staff that were interviewed
Provider Role	Describes the role of the family planning provider (e.g., staff, resident, house officer).
Training Exposure	Mentions of exposure to or training in family planning during medical education or professional development
Current FP Practices	Describes how family planning services are currently delivered by the provider or facility
Contraceptive Use	Refers to patterns or prevalence of contraceptive use in the population being served
Contraceptive Methods	Types of contraceptive methods mentioned (e.g., pills, IUDs, implants, condoms)
Motivations	Discussion of motivations for using contraception in general, as well as motivations for preferring/using specific contraceptive methods
Barriers to Access to FP Services	Identified or hypothetical barriers for youth accessing family planning services in Tamale. This code should be used concurrently with other codes specifying the content of the barrier
Facilitators to Access to FP Services	Identified or hypothetical facilitators for youth accessing family planning services in Tamale. This code should be used concurrently with other codes specifying the content of the facilitator
Logistics	Discussion of the practical aspects of accessing family planning services, including monetary cost, transportation, and proximity to clinics or pharmacies
Health Systems	Discussion of organization of healthcare facilities providing family planning services, including clinic hours, staff behavior, service quality, wait times, and privacy or confidentiality
Contraceptive availability	The availability of contraceptive methods at various locations such as clinics, pharmacies, community distribution points, or outreach programs
Knowledge	General awareness or understanding of family planning methods and services, including sources of knowledge (peers, social media, school, etc.)
Religion	Discussion of religious beliefs or teachings as related to the use of family planning.
Misconceptions	Discussion of myths or misinformation, such as beliefs that contraception causes infertility or weight gain
Societal Attitudes	Community beliefs and norms, including stigma, judgment, and gender roles, that affect acceptance and use of family planning
Partner Attitudes	Behaviors and beliefs from romantic or sexual partners that influence family planning use
Parental Attitudes	Beliefs and reactions from parents, including opposition or support, that affect youth access to and use of family planning
Peer Attitudes	Beliefs and reactions from peers, including opposition or support, that affect youth access to and use of family planning
Healthcare provider attitudes	Beliefs, biases, and behaviors of healthcare workers that affect youth engagement with family planning services
Side Effects	Experiences or concerns of side effects from contraceptive methods (e.g., irregular periods, weight gain)
Decision-making	Discussion of how decisions about using family planning services are made, including who is involved and the factors influencing the choices.
Personal	Decisions made independently by the youth
External	Decisions influenced or made by others such as partners, family members, or community, affecting the individual’s use of family planning
P2P Counseling Specific	Discussion related to P2P Counseling Program. This code should be used concurrently with other codes relating to the content of the text
Youth Friendly Counseling Program Implementation	
Counseling Content	The topics/information included in counseling sessions (e.g., contraceptive options, side effects, sexual health, etc.)
Mode of Delivery	The method by which counseling is provided, such as one-on-one, group sessions, digital platforms, outreach groups, etc.
Setting of Counseling	The physical or virtual location where counseling takes place, such as clinics, schools, community centers, town, or online
Availability of Services	The extent to which youth-friendly counseling programs are accessible, including hours of operation, staffing, multiple locations, and outreach efforts
Counselor Characteristics	Desired traits, identity, and background of effective FP counselors
Roles of a Peer Counselor	Key responsibilities peer counselors take on in delivering family planning services, such as providing emotional/logistic support, knowledge, method provision
Suggested Changes to P2P Program	Ideas to enhance the structure, content, or delivery of the proposed peer-to-peer family planning program
Peer Counselor Training	Descriptions of how peer counselors should be trained, who they are trained by, etc.
Great Quote	Used to highlight notable quotes within the interview that illustrate a specific code or theme in a particularly expressive or impactful way. Should be applied concurrently with other relevant codes

Youth Interview Codebook

**Code**	**Definition**
Interviewee Characteristics	Characteristics of the youth that was interviewed (age, gender, schooling, living situation, etc.)
Personal Experiences with Family Planning	Includes any mention by youth of their own use or non-use of family planning methods. This code captures direct personal experience
Peer Experiences with Family Planning	Includes any mention by youth of their knowledge of peers’ use. This code captures indirect awareness through friends who have used family planning methods
Contraceptive Use	Refers to patterns or prevalence of contraceptive use in the population being served
Contraceptive Methods	Types of contraceptive methods mentioned (e.g., pills, IUDs, implants, condoms)
Motivations	Discussion of motivations for using contraception in general, as well as motivations for preferring/using specific contraceptive methods
Barriers to Access to FP Services	Identified or hypothetical barriers for youth accessing family planning services in Tamale. This code should be used concurrently with other codes specifying the content of the barrier
Facilitators to Access to FP Services	Identified or hypothetical facilitators for youth accessing family planning services in Tamale. This code should be used concurrently with other codes specifying the content of the facilitator
Logistics	Discussion of the practical aspects of accessing family planning services, including monetary cost, transportation, and proximity to clinics or pharmacies
Health Systems	Discussion of organization of healthcare facilities providing family planning services, including clinic hours, staff behavior, service quality, wait times, and privacy or confidentiality
Contraceptive availability	The availability of contraceptive methods at various locations such as clinics, pharmacies, community distribution points, or outreach programs
Knowledge	General awareness or understanding of family planning methods and services, including sources of knowledge (peers, social media, school, etc.)
Religion	Discussion of religious beliefs or teachings as related to the use of family planning
Misconceptions	Discussion of myths or misinformation, such as beliefs that contraception causes infertility or weight gain
Societal Attitudes	Community beliefs and norms, including stigma, judgment, and gender roles, that affect acceptance and use of family planning
Partner Attitudes	Behaviors and beliefs from romantic or sexual partners that influence family planning use
Parental Attitudes	Beliefs and reactions from parents, including opposition or support, that affect youth access to and use of family planning
Peer Attitudes	Beliefs and reactions from peers, including opposition or support, that affect youth access to and use of family planning
Healthcare provider attitudes	Beliefs, biases, and behaviors of healthcare workers that affect youth engagement with family planning services
Side Effects	Experiences or concerns of side effects from contraceptive methods (e.g., irregular periods, weight gain)
Decision-making	Discussion of how decisions about using family planning services are made, including who is involved and the factors influencing the choices
Personal	Decisions made independently by the youth
External	Decisions influenced or made by others such as partners, family members, or community, affecting the individual’s use of family planning
P2P Counseling Specific	Discussion related to P2P Counseling Program. This code should be used concurrently with other codes relating to the content of the text
Youth Friendly Counseling Program Implementation	
Counseling Content	The topics/information included in counseling sessions (e.g., contraceptive options, side effects, sexual health, etc.)
Mode of Delivery	The method by which counseling is provided, such as one-on-one, group sessions, digital platforms, outreach groups, etc.
Setting of Counseling	The physical or virtual location where counseling takes place, such as clinics, schools, community centers, town, or online
Availability of Services	The extent to which youth-friendly counseling programs are accessible, including hours of operation, staffing, multiple locations, and outreach efforts
Counselor Characteristics	Desired traits, identity, and background of effective FP counselors
Roles of a Peer Counselor	Key responsibilities peer counselors take on in delivering family planning services, such as providing emotional/logistic support, knowledge, method provision
Suggested Changes to P2P Program	Ideas to enhance the structure, content, or delivery of the proposed peer-to-peer family planning program
Peer Counselor Training	Descriptions of how peer counselors should be trained, who they are trained by, etc.
Great Quote	Used to highlight notable quotes within the interview that illustrate a specific code or theme in a particularly expressive or impactful way. Should be applied concurrently with other relevant codes

## References

[1] United National Population Fund Explainer: How Does Family Planning Save Lives? 2025. https://www.unfpa.org/news/explainer-how-does-family-planning-save-lives.

[2] World Health Organization (2025). Family Planning/Contraception Methods. https://www.who.int/news-room/fact-sheets/detail/family-planning-contraception.

[3] FP2030 (2012). Family Planning 2030. The FP2030 Strategy. https://www.fp2030.org/fp2030-strategy-document/.

[4] Starrs A.M., Ezeh A.C., Barker G., Basu A., Bertrand J.T., Blum R., Coll-Seck A.M., Grover A., Laski L., Roa M. (2018). Accelerate progress—Sexual and reproductive health and rights for all: Report of the Guttmacher–Lancet Commission. The Lancet.

[5] United Nations Department of Economic and Social Affairs, United Nations Sustainable Development (2015). THE 17 GOALS|Sustainable Development. https://sdgs.un.org/goals.

[6] United Nations Population Fund (1994). International Conference on Population and Development (ICPD). https://www.unfpa.org/events/international-conference-population-and-development-icpd.

[7] (2026). Focus 2030. The Access to Contraception Around the World: Situational Analysis and Current Challenges. https://focus2030.org/en/the-access-to-contraception-around-the-world-situational-analysis-and-current-challenges/.

[8] Ba D.M., Ssentongo P., Agbese E., Kjerulff K.H. (2019). Prevalence and predictors of contraceptive use among women of reproductive age in 17 sub-Saharan African countries: A large population-based study. Sex. Reprod. Healthc..

[9] Mccurdy R.J., Schnatz P.F., Weinbaum P.J., Zhu J. (2014). Contraceptive use in adolescents in Sub-Saharan Africa: Evidence from Demographic and Health Surveys. Conn. Med..

[10] Ghana Statistical Service, Demographic and Health Surveys Program (ICF) (2023). Ghana Demographic and Health Survey 2022.

[11] World Health Organization (2019). Contraception: Evidence Brief. Contraception Enables People to Make Informed Choices About Their Sexual and Reproductive Health.

[12] Keogh S.C., Otupiri E., Castillo P.W., Li N.W., Apenkwa J., Polis C.B. (2021). Contraceptive and abortion practices of young Ghanaian women aged 15–24: Evidence from a nationally representative survey. Reprod. Health.

[13] Ninsiima L.R., Chiumia I.K., Ndejjo R. (2021). Factors influencing access to and utilisation of youth-friendly sexual and reproductive health services in sub-Saharan Africa: A systematic review. Reprod. Health.

[14] Nartey E.B., Babatunde S., Okonta K.E., Kotoh A.M., Amoadu M., Abraham S.A., Agyare D.F., Baah J.A., Obeng P. (2025). Prevalence and barriers to the utilization of adolescent and youth-friendly health services in Ghana: Systematic review and meta-analysis. Reprod. Health.

[15] Asare B.Y.A., Aryee S.E., Kotoh A.M. (2020). Sexual behaviour and the utilization of youth friendly health Services: A cross-sectional study among urban youth in Ghana. Int. J. Afr. Nurs. Sci..

[16] Obeng A.A., Blumenberg C., Afagbedzi S.K., Wado Y.D., Nilsen K. (2025). Demand for family planning satisfied by modern methods in Ghana: Trends and inequalities (2013–2022). BMC Public Health.

[17] Nyarko S.H., Sparks C.S., Bitew F. (2019). Spatio-temporal variations in unmet need for family planning in Ghana: 2003–2014. Genus.

[18] Hernandez S., Quinteros Baumgart C., Tolleson K., Malechi H., Ayete Labi A., Imogie S., Sison R., Brault M.A., Charadan A.M.S. (2026). Enhancing family planning services in northern Ghana: A landscape assessment to develop strategic interventions using an academic partnership approach. medRxiv.

[19] Wichterle K., Hernandez S., Tolleson K., Brault M.A., Caesar A.A., Ayete-Labi A., Imogie S.A., Yakubu M.S., Opare-Duodo J., Sison R. (2026). Adapting and Implementing a Youth-Friendly Sexual and Reproductive Health Counseling Training for Providers in Northern Ghana: A Pilot Study of Acceptability, Appropriateness, and Feasibility. BMC Glob. Health.

[20] Bakesiima R., Beyeza-Kashesya J., Tumwine J.K., Chalo R.N., Gemzell-Danielsson K., Cleeve A., Larsson E.C. (2021). Effect of peer counselling on acceptance of modern contraceptives among female refugee adolescents in northern Uganda: A randomised controlled trial. PLoS ONE.

[21] Flanagan S., Gorstein A., Nicholson M., Bradish S., Amanyire D., Gidudu A., Aucur F., Twesigye J., Kyateka F., Balamaga S. (2021). Behavioural intervention for adolescent uptake of family planning: A randomized controlled trial, Uganda. Bull. World Health Organ..

[22] Wondimagegene Y.A., Debelew G.T., Koricha Z.B. (2023). Effectiveness of peer-led education interventions on contraceptive use, unmet need, and demand among adolescent girls in Gedeo Zone, South Ethiopia. A cluster randomized controlled trial. Glob. Health Action.

[23] Society for Adolescent Health and Medicine (2025). Guidelines on the Inclusion and Protection of Adolescent Minors and Young Adults in Health Research: A Position Statement of the Society for Adolescent Health and Medicine. J. Adolesc. Health.

[24] English A., Ford C.A., Kahn J.A., Kharbanda E.O., Middleman A.B., Society for Adolescent Health and Medicine (2013). Adolescent consent for vaccination: A position paper of the Society for Adolescent Health and Medicine. J. Adolesc. Health.

[25] English A., Ford C.A. (2022). Adolescent Consent and Confidentiality: Complexities in Context of the 21st Century Cures Act. Pediatrics.

[26] Kreniske P., Hoffman S., Ddaaki W., Nakyanjo N., Spindler E., Ssekyewa C., Isabirye D., Nakubulwa R., Proscovia N., Daniel L. (2023). Capacity to Consent to Research Among Adolescent-Parent Dyads in Rakai, Uganda. J. Pediatr..

[27] Liu C., Cox R.B., Washburn I.J., Croff J.M., Crethar H.C. (2017). The Effects of Requiring Parental Consent for Research on Adolescents’ Risk Behaviors: A Meta-analysis. J. Adolesc. Health.

[28] Day S., Kapogiannis B.G., Shah S.K., Wilson E.C., Ruel T.D., Conserve D.F., Strode A., Donenberg G.R., Kohler P., Slack C. (2020). Adolescent HIV Research Ethics in Resource-Constrained Settings: Low and Middle-Income Country Research Consortium Experience and Scoping Review. Lancet HIV.

[29] Loveday M., Goga A., Dhai A., Labuschaigne M., Roussouw T., Burgess T., Strode A., Wallace M., Blockman M., Daniels B. (2022). Ethically acceptable consent approaches to adolescent research in South Africa. South. Afr. J. HIV Med..

[30] Nkosi B., Zanoni B., Seeley J., Strode A. (2022). The ethical-legal requirements for adolescent self-consent to research in sub-Saharan Africa: A scoping review. Bioethics.

[31] Santelli J.S., Smith Rogers A., Rosenfeld W.D., DuRant R.H., Dubler N., Morreale M., English A., Lyss S., Wimberly Y., Schissel A. (2003). Guidelines for adolescent health research. A position paper of the Society for Adolescent Medicine. J. Adolesc. Health.

[32] Michie S., van Stralen M.M., West R. (2011). The behaviour change wheel: A new method for characterising and designing behaviour change interventions. Implement. Sci..

[33] Sidamo N.B., Meskele M., Hebo S.H., Teshome A., Oumer B., Ibrahim Y., Yihune M., Mitikie G., Agegnehu Y. (2026). Co-Designing an Integrated Intervention Package to Strengthen Adolescent Sexual and Reproductive Health Services in Primary Healthcare Units in Southern Ethiopia: A Human-Centered Design Approach. medRxiv.

[34] Gerchow L., Lanier Y., Fayard A.L., Squires A. (2024). Cocreating First Steps, a Toolkit to Improve Adolescent Sexual and Reproductive Health Services: Qualitative Human-Centered Design Study With Hispanic and Black Adolescent Mothers in New York City. JMIR Pediatr. Parent..

[35] Meherali S., Rehmani A.I., Ahmad M. (2025). Adolescent Voices in Action—Co-Designing Digital Sexual and Reproductive Health Knowledge Translation Interventions: Community-Based Participatory Action Project. JMIR Human. Factors.

[36] Fakoya I., Cole C., Larkin C., Punton M., Brown E., Ballonoff Suleiman A. (2022). Enhancing Human-Centered Design With Youth-Led Participatory Action Research Approaches for Adolescent Sexual and Reproductive Health Programming. Health Promot. Pract..

[37] Abdulai A.M., Iddrisu A., Monne R., Nabirkampitib M., Djangbah D., Mustapha A.L., Kpiinfaa T.N. (2023). Assessing the Availability and Utilisation of Adolescent Reproductive Health Services in Northern Region of Ghana. Texila Int. J. Public Health.

[38] Amoah E.J., Hinneh T., Aklie R. (2023). Determinants and prevalence of modern contraceptive use among sexually active female youth in the Berekum East Municipality, Ghana. PLoS ONE.

[39] Apambila R.N., Owusu-Asubonteng G., Dassah E.T. (2020). Contraceptive use among young women in northern Ghana: A community-based study. Eur. J. Contracept. Reprod. Health Care.

[40] Munakampe M.N., Zulu J.M., Michelo C. (2018). Contraception and abortion knowledge, attitudes and practices among adolescents from low and middle-income countries: A systematic review. BMC Health Serv. Res..

[41] Decker M.R., Wood S.N., Byrne M.E., Yao-N’dry N., Thiongo M., Gichangi P., OlaOlorun F.M., Koffi A.K., Radloff S., Ahmed S. (2021). Gendered power dynamics and threats to sexual and reproductive autonomy among adolescent girls and young adult women: A cross-sectional survey in three urban settings. PLoS ONE.

[42] Desai S., Pandey N., Singh R.J., Bhasin S. (2021). Gender inequities in treatment-seeking for sexual and reproductive health amongst adolescents: Findings from a cross-sectional survey in India. SSM Popul. Health.

[43] Heise L., Greene M.E., Opper N., Stavropoulou M., Harper C., Nascimento M., Zewdie D., Darmstadt G.L., Greene M.E., Hawkes S. (2019). Gender inequality and restrictive gender norms: Framing the challenges to health. Lancet.

[44] Ogala D.E., Xiang X. (2026). Gender power dynamics and contraceptive decision-making among sexually active girls and young women in Nigeria. Cult. Health Sex..

[45] Tesha J., Fabian A., Mkuwa S., Misungwi G., Ngalesoni F. (2023). The role of gender inequities in women’s access to reproductive health services: A population-level study of Simiyu Region Tanzania. BMC Public Health.

[46] Biney A.A.E., Wright K.J., Kushitor M.K., Jackson E.F., Phillips J.F., Awoonor-Williams J.K., Bawah A.A. (2021). Being ready, willing and able: Understanding the dynamics of family planning decision-making through community-based group discussions in the Northern Region, Ghana. Genus.

[47] Loll D., Fleming P.J., Manu A., Morhe E., Stephenson R., King E.J., Hall K.S. (2019). Reproductive Autonomy and Modern Contraceptive Use at Last Sex Among Young Women in Ghana. Int. Perspect. Sex. Reprod. Health.

[48] Sulemana I., Gbeti C., Dalaba M., Yidana A., Aninanya G.A. (2025). Determinants of family planning services uptake among women within the reproductive age in the Yendi municipality in Northern Ghana. BMC Public Health.

[49] Ziblim S.D., Suara S.B., Adam M. (2022). Sexual behaviour and contraceptive uptake among female adolescents (15–19 years): A cross-sectional study in Sagnarigu Municipality, Ghana. Ghana J. Geogr..

[50] Obiezu-Umeh C., Nwaozuru U., Mason S., Gbaja-Biamila T., Oladele D., Ezechi O., Iwelunmor J. (2021). Implementation Strategies to Enhance Youth-Friendly Sexual and Reproductive Health Services in Sub-Saharan Africa: A Systematic Review. Front. Reprod. Health.

[51] Denno D.M., Hoopes A.J., Chandra-Mouli V. (2015). Effective strategies to provide adolescent sexual and reproductive health services and to increase demand and community support. J. Adolesc. Health.

[52] George A.S., Amin A., de Abreu Lopes C.M., Ravindran T.K.S. (2020). Structural determinants of gender inequality: Why they matter for adolescent girls’ sexual and reproductive health. BMJ.

[53] World Health Organization WHO Recommendations on Adolescent Sexual and Reproductive Health and Rights. https://www.who.int/publications/i/item/9789241514606.

[54] Svanemyr J., Amin A., Robles O.J., Greene M.E. (2015). Creating an Enabling Environment for Adolescent Sexual and Reproductive Health: A Framework and Promising Approaches. J. Adolesc. Health.

[55] Sulemana Z., Gqunu S., Abobo F., Halm H., Awuku N.O., Kumi R., Amoore B.Y., Ephraim R.K.D., Duah E., Agoni C. (2024). Knowledge and utilization of family planning services among tertiary students in Northern Ghana: The case of College of Nursing and Midwifery, Nalerigu. Afr. J. Reprod. Health.

[56] Kyilleh J.M., Tabong P.T.N., Konlaan B.B. (2018). Adolescents’ reproductive health knowledge, choices and factors affecting reproductive health choices: A qualitative study in the West Gonja District in Northern region, Ghana. BMC Int. Health Hum. Rights.

